# Supplementary material for: Noradrenergic infraslow rhythm during sleep is the critical link between heart-rate dynamics and memory consolidation
Source: eLife. 2026 Aug 28;15:RP110252. doi: 10.7554/eLife.110252 (PMC13524380; doi:10.7554/eLife.110252)
Supplement: Peer review history

# Editor report: eLife Assessment

**DOI:** 10.7554/eLife.110252.3.sa0

**Authors:** Adrien Peyrache

**Affiliations:** McGill University Canada

**Keywords:** Convincing, Important

This **important** study provides evidence that locus coeruleus activity is coordinated with heart rate during sleep, confirming previous work in mice and humans, with a possible role for sleep-dependent memory consolidation. The claims are supported by **convincing** evidence. This work will be of interest to neuroscientists focusing on sleep, memory, and autonomic functions.

# Referee report: Reviewer #2 (Public review)

**DOI:** 10.7554/eLife.110252.3.sa1

Summary:

This convincing study builds on previously published findings in both mice and humans to advance quantitative insights into the coupling between noradrenergic activity fluctuations during mouse NREM sleep and heart rate fluctuations. The work reaffirms the presence of coordinated infraslow fluctuations in sigma power and heart rate during NREM sleep and that this coordination is enabled by noradrenaline-releasing neurons in the locus coeruleus. Also supporting previously published work in mice and humans, the authors describe a link between the strength of these infraslow fluctuations and memory consolidation in mice and humans.

Strengths:

A major finding of this study is the mechanistic insight it provides into the regulation of the previously understudied very-low-frequency (0-0.15 Hz) component of heart rate variability, and the demonstration, through elegant optogenetic bidirectional interference, that infraslow noradrenergic fluctuations are an underlying driving force. This finding will promote recognition of heart rate variability in sleeping mice as a read-out of neuronal activity patterns that control autonomic balance.

Another strength of the study is its translational part, whereby the sigma power-heart rate coupling in mouse is used to identify a previously unrecognized correlation between such coupling and memory consolidation in humans. This widens the applicability of heart rate variability measures, highlighting their use as biomarkers for noradrenergic fluctuations and associated sleep-dependent memory consolidation.

Weaknesses:

The study impresses by the thorough parallel analysis of both mouse and human correlational data between electrophysiological and fluorescent activity measures of the sleeping brain. Further work will be needed to disentangle the mechanisms by which heart rate is regulated, notably the contribution of parasympathetic and sympathetic nervous systems, to establish the very low frequency heart rate variability in mice as a novel biomarker for noradrenergic dynamics in the sleeping brain.

# Author comment: Author response

**DOI:** 10.7554/eLife.110252.3.sa2

**Authors:** Sofie Jacobsen, Allison B Morehouse, Pin-chun Chen, Yi Qian, Ryszard Stefan Gomolka, Mie Andersen, Maiken Nedergaard, Sara Mednick, Celia Kjaerby

**Affiliations:** University of Copenhagen Copenhagen Denmark; University of California, Irvine Irvine United States; University of Oxford Oxford United Kingdom; University of Copenhagen Copenhagen Denmark; University of Copenhagen Copenhagen Denmark; University of Copenhagen Copenhagen Denmark; University of Copenhagen Copenhagen Denmark; University of California, Irvine Irvine United States; University of Copenhagen Copenhagen Denmark

The following is the authors’ response to the original reviews.

**Public Reviews:**

**Reviewer #1 (Public review):**

Summary:

This study examined whether infraslow fluctuations in noradrenaline and in heart rate are coupled and how they are affected by sleep transitions. The authors used the fluorescent NA biosensor GRAB-NE2m in the medial prefrontal cortex of mice to record extracellular NA while also recording EEG and EMG during sleep-wake episodes. They also analyzed previously published human data to reproduce relationships they found between sigma power and RR intervals in mice.

Strengths:

This is an impressive study with significant strengths, as it involves a rich set of data that includes not only observations of associations between heart rate and noradrenergic dynamics but also optogenetic manipulation of the locus coeruleus. Human data is presented to show parallels in the association between sigma power during sleep and phasic heart-rate bursts.

We thank Reviewer #1 for their thoughtful, detailed, and constructive evaluation of our manuscript. We appreciate their recognition of the strengths of the study, particularly the integration of noradrenergic recordings, optogenetic manipulation, and cross-species analyses. We are especially grateful for the reviewer’s careful attention to clarity, experimental interpretation, and control comparisons. The comments have helped us sharpen the framing of our hypotheses, clarify causal claims, improve statistical reporting, and better explain our closed-loop approach and heart rate analyses. We have addressed each point in detail below and believe that the revisions substantially strengthen the manuscript.

Weaknesses:

(1) Language could be clearer and more precise. As detailed below, in both the introduction and the discussion, the way the hypotheses and study objectives are described could use some revision to be more precise and accurate.

Thank you for this helpful comment. We have sharpened the description of the study objectives, hypotheses, and interpretation of the findings to better distinguish between what was directly tested, what was inferred, and what remains speculative. We revised the language throughout these sections to improve clarity, accuracy, and overall readability

(1A) In the introduction on p. 4: The overarching question is framed as "could the peripheral autonomous systems be a read-out of the central LC-NE system and thus be a biomarker of memory consolidation and LC dysfunction?" This gives the impression that the LC function would be the main influence on peripheral autonomous systems. There are, of course, many influences on peripheral autonomous systems, so it would be advisable for the authors to be more specific here about what signal(s) in particular would be predicted to be sensitive markers of LC function.

Thank you for this important point. We agree that heart rate reflects the integrated output of multiple autonomic mechanisms and should not be interpreted as being exclusively driven by LC activity. Cardiac dynamics arise from the balance between sympathetic and parasympathetic influences, which themselves are regulated by several central and peripheral systems. In addition, recent work shows that the infraslow oscillations observed during NREM sleep are not restricted to norepinephrine alone but also involve other neuromodulatory systems, including acetylcholine and serotonin (e.g., Teng et al., PNAS 2025, Kjaerby et al., iScience, 2026). Our intention was therefore not to imply that the LC is the sole driver of peripheral autonomic dynamics. We have revised our overarching questions to make them more specific: Please see new text below:

(Introduction, page 4/5). “The sympathetic and parasympathetic autonomic nervous system are involved in HRV, which is conventionally analyzed across three primary frequency bands: high frequency (HF) HRV, low frequency (LF) HRV, and very low frequency (VLF) HRV (Berntson et al., 1997). Due to the frequency overlap with VLF HRV, we wondered if central infraslow NE dynamics could be linked to this poorly understood HRV indicator. Furthermore, are infraslow NE fluctuations directly reflected by HRV under different physiological states or does LC–HR coupling scale differently with LC output? Specifically, if infraslow NE oscillations display faster frequencies - as occurs during sleep fragmentation - will cardiac dynamics exhibit corresponding changes? Conversely, given that stronger infraslow NE dynamics correlate with memory consolidation through their regulation of sleep spindles, could the peripheral autonomic signatures provide an accessible cross-species biomarker of spindle-dependent memory consolidation? Addressing these questions could help bridge mechanistic insights into LC-mediated sleep regulation with established HRV metrics used in human physiology.”

(1B) In the discussion on p. 12: "In this study, we leveraged real-time measurements of mPFC NE levels and HR measurements from EMG recordings in mice to investigate the causal link between the two variables with high temporal resolution in freely moving sleeping mice, with similar inspection in humans." To test the causal link between mPFC NA levels and HR measures, the study would manipulate NA levels just in the mPFC and not elsewhere in the brain. However, in this study, the manipulation occurred in the LC, and so there would be broad cortical changes in NA levels. Thus, it could be that LC activity causes HR changes via a non-PFC pathway.

We thank the reviewer for this important comment. Indeed, mPFC NE is merely a readout of LC activations and we expect that NE in other brain regions would show the same patterns. Indeed, mPFC NE is not expected to provide any causal link to heart rate. We have revised added a sentence to the results section and also changed the initial summary part of the discussion to reflect this better.

(Results, page 6). “mPFC was selected as a representative cortical readout of LC-mediated norepinephrine dynamics, as infraslow NE fluctuations are coordinated across widespread brain regions.”

(Discussion, page 14/15). “Variability in HR is a non-invasive biomarker of autonomic nervous system function and is frequently disrupted in ageing and Alzheimer’s disease. Here, by combining real-time measurements of mPFC NE dynamics with simultaneous HR recordings in freely sleeping mice, we demonstrate that HR closely tracks the infraslow phasic activity of the LC–NE system.”

(2) Comparisons with the control condition need further development.

(2A) While the authors did include a key YFP control condition, in the main text no direct statistical comparison between the closed-loop optogenetic stimulation (ChR2) condition and the YFP control condition was reported. (It was reported in Supplementary Figure 2c-d.) Instead, in the main text, the authors only reported that the effects of stimulation were significant in the closed-loop condition and not in the control. However, that is not the same as demonstrating that the two conditions significantly differed from each other, and it is the direct test that is important for the conclusions, so it seems important to include this result in the main presentation.

We thank the reviewer for this important point and agree that direct statistical comparisons between ChR2 and YFP conditions are important for interpretation. These comparisons were performed and are shown in Supplementary Figure 2c–d, but we acknowledge that this was not sufficiently emphasized in the main text. We are now more clearly referring to this comparison in Result section:

(Results, page 9). “The magnitude of pre-stimulation NE descent and post-stimulation NE ascent was reduced as the thresholds increased, indicating less pronounced NE dynamics as LC stimulation became more frequent (Fig. 2e, for direct comparison with YFP control, see Suppl. Fig. 2c-d).”

Our rationale for prioritizing the within-animal threshold comparisons in the main figure was that the central experimental question concerned how progressive shifts in infraslow NE oscillatory frequency influence the NE–HR relationship. Because variability in viral expression levels (both NE sensor expression and LC opsin expression) introduces substantial between-animal variability, we considered within-animal comparisons across threshold conditions to provide the most informative representation of how changes in LC-driven NE dynamics alter cardiac responses. That said, we agree that highlighting the direct ChR2 versus YFP comparison is important for the overall interpretation. As mentioned, the reference to Suppl. Fig. 2c– d, where the between-group analyses are visualized are now clearly referred to.

(2B) In addition, the authors should address the issue that the pre-stimulation NE was consistently significantly lower in the YFP condition than in the ChR2 condition (see Supplementary Figure 2c), which is a potential confound.

We thank the reviewer for bringing up this important point. We agree that differences in stimulation timing between ChR2 and YFP animals could complicate interpretation in a closed-loop design and appreciate the opportunity to clarify this aspect of the experiment.

In the ChR2 condition, animals were exposed to repeated optogenetic LC activation designed to mimic progressively faster infraslow NE dynamics. Such repeated stimulation is expected to produce a gradual elevation in tonic NE levels across the recording session, which explains the higher pre-stimulation baseline relative to YFP controls. We acknowledge that elevated tonic NE levels could introduce additional physiological effects. For example, higher NE tone would be expected to increase α2mediated autoinhibitory feedback on LC neurons and presynaptic NE release. Within the LC itself, we expect that optogenetic stimulation would largely override such effects due to the strong Na+-mediated depolarization induced by ChR2 activation. However, NE release in downstream regions such as the mPFC may be influenced to some extent by elevated tonic noradrenergic tone. Importantly, such feedback mechanisms would likely also occur under physiological conditions characterized by elevated LC activity, such as stress or sleep fragmentation. Because the goal of our stimulation paradigm was to model progressively faster infraslow NE dynamics under physiologically relevant conditions, we believe this feature of the manipulation may in fact increase the translational relevance of the model. We specifically address this elevation in Figure 3, where we show that very rapid stimulation regimes are accompanied by signs of compensatory cardiovascular regulation, likely reflecting baroreceptor-mediated responses to sustained increases in heart rate.

We agree that the precise contribution of elevated tonic NE to the overall manipulation cannot be fully disentangled in the present study. We therefore avoid overinterpreting these effects and have instead added text to the manuscript acknowledging this consideration without extensive speculation.

(Results, page 9) “Since pre-stimulation NE baseline levels progressively became higher in the ChR2 condition compared with YFP controls (Fig. 2c+e, Supplementary Fig. 3a-f), it demonstrates that they arise from stimulation-dependent modulation of noradrenergic tone rather than nonspecific signal drift. As a result, the pre-stimulation state at higher thresholds differed between ChR2 and control conditions, which should be considered when interpreting the immediate effects of laser stimulation across thresholds.”

(2C) Direct comparison of the strengths of correlations shown in Figure 2h vs. Supplementary Figure 2f should be included. Currently, we see relatively weak correlations in both ChR2 and YFP conditions, and it is not clear if the relationships differ in the control. It seems they are still present in the control condition but weaker which would contradict the apparently broad claim on p. 7 that "No such effects were present in the control condition" (it is not entirely clear whether this claim refers to all effects discussed in the figure or just a subset - this language should be clarified).

We thank the reviewer for this important comment and agree that the original wording could be interpreted as implying a complete absence of an NE–RR relationship in the YFP condition. To address this concern, we directly compared the strength of the NE– RR relationship between ChR2 and YFP animals. Please see Supplementary Figure 3h.

Using a linear mixed-effects model that accounted for repeated measurements within animals, we found that the slope of the NE–RR relationship was significantly steeper in ChR2 animals than in YFP controls (all thresholds: slope difference = 1.915, *p* < 0.0001; thresholds −15, −10, and −5 only: slope difference = 2.367, *p* < 0.0001). Consistent with this result, comparison of Pearson correlations using Fisher's r-to-z transformation also indicated significantly stronger coupling in ChR2 animals than in YFP controls (see Statistics in the Supplementary File).

These analyses demonstrate that an inverse relationship between NE and RR is present under physiological conditions in YFP animals, but that optogenetic LC activation substantially strengthens this coupling. We have revised the corresponding text to clarify this:

(Results, Page 9): “An inverse NE–RR relationship was present in both ChR2 (Fig. 2h) and YFP (Suppl. Fig. 3f) groups but was significantly stronger in ChR2 animals than in YFP controls (Suppl. Fig. 3h).”

(2D) Did the YFP controls vs. ChR2 animals show any differences in the number of NA states that triggered stimulation in the closed-loop system? With ChR2 animals, stimulation changes NA, which could change future triggering. In YFP animals, nothing changes NA (other than natural fluctuations), so the dynamics of stimulation timing could diverge between groups in a way that complicates interpretation. Specifically, if ChR2 stimulation raises NA and prevents future threshold crossings, ChR2 animals may end up receiving fewer subsequent stimulations than YFP animals (or a different temporal clustering). If the number or pattern of stimulation differed in two groups, it would be important to have a yoked control where matched animals get the same stimulation pattern but not triggered by their own NA.

We thank the reviewer for this important point. We agree that differences in stimulation timing between ChR2 and YFP animals could complicate interpretation in a closed-loop design.

Importantly, stimulation triggering was based on relative declines in NE fluorescence calculated against a rolling 2-minute baseline, rather than absolute NE levels. Thus, as tonic NE levels gradually increased in ChR2 animals, the threshold adapted accordingly, reducing the likelihood that elevated baseline NE alone would prevent future triggering. Instead, stimulation continued to occur when NE declined relative to the recent baseline, thereby preserving the infraslow closed-loop structure.

The number of stimulation events across thresholds is already reported in the manuscript (Methods, p. 30 and corresponding figure legends), but we have now indicated more clearly in the result section where to find the information:

(Results, Page 9). “Mean traces of NE and RR were aligned to LC stimulation onset (Fig. 2c-d, for number of laser stimulations see Fig. 2 legend or Methods).”

(Methods, Page 31). “For the LC activation-related analysis, 108 events were found for Threshold -15 (11 of these being YFP), 260 events for Threshold -10 (55 of these YFP), 777 events for Threshold -5 (296 of these YFP), 1,444 events for Threshold 0 (510 of these YFP), and 1,148 events for Threshold 5 (377 of these YFP) across ten animals (four being YFP).”

While the total number of events was lower in YFP animals, this is expected in part due to the smaller group size (4 YFP vs. 6 ChR2 animals included in this analysis). Furthermore, because ChR2 stimulation increased NE levels by design, more pronounced subsequent declines in NE may have modestly facilitated additional threshold crossings.

Nevertheless, the overall temporal structure of the stimulation paradigm remained comparable across groups, and YFP animals underwent the same closed-loop stimulation protocol. Our primary comparison was mainly based on shifts in within-animal NE oscillatory frequency and how this change would impact the connection to HR. Thus, we believe the present control condition appropriately addresses the central question of whether optogenetic LC activation are able to conduct a continuum of NE oscillations.

(3) Some more discussion/explanation of the rationale for the closed-loop approach and how it influences how we should interpret the results could be useful. For instance, currently, it is not clear whether LC stimulation needs to be timed after an NA dip to yield the effects seen.

We thank the reviewer for pointing this out. The rationale for the closed-loop LC stimulation approach was to test whether the relationship between infraslow LC–NE dynamics and heart rate is maintained only under physiological infraslow conditions or whether it breaks down when the rhythm becomes progressively faster, as occurs during sleep fragmentation and other high-arousal states. Specifically, we asked whether heart rate continues to track LC–NE fluctuations as the infraslow rhythm shifts toward higher frequencies, thereby assessing its utility as a potential biomarker of disrupted restorative sleep.

To address this while preserving the intrinsic temporal structure of infraslow LC activity, we implemented a closed-loop strategy in which stimulations were triggered following defined declines in the NE signal, using a rolling preceding 2-minute window as baseline. This allowed LC activation to occur during the descending phase of the endogenous infraslow cycle, maintaining its physiological phase structure while systematically increasing its effective frequency. By progressively relaxing the decline threshold, stimulations were triggered earlier in the cycle, thereby compressing the infraslow period in a controlled manner.

Importantly, the intention was not to test whether LC stimulation specifically needs to occur after an NE dip to elicit the observed effects. Rather, triggering stimulation during the decay phase provided a way to accelerate the infraslow rhythm without disrupting sleep through indiscriminate stimulation. This enabled us to examine whether the coupling between LC–NE dynamics and heart rate remains stable under increasingly rapid infraslow regimes. Our results indicate that this relationship weakens at higher infraslow stimulation frequencies, suggesting that heart rate reliably reflects physiological LC–NE oscillations but becomes less tightly coupled when the rhythm is compressed beyond its normal range.

We have clarified this rationale in the revised manuscript by adding the below section in the result section.

(Result, page 8/9). “This approach enabled controlled compression of the infraslow NE cycle by triggering LC activation during the descending phase of the endogenous NE signal, thereby increasing the effective oscillatory frequency while preserving the temporal structure of physiological LC–NE dynamics. This strategy allowed us to test whether heart-rate responses continue to track LC-driven NE fluctuations as the infraslow rhythm becomes progressively faster.”

(4) The section on heart rate decelerations is hard to follow. In particular, I was not sure how to interpret Figure 3f-j. For Figure 3f, what does the middle line represent? The laser onset or the max RR value after laser onset? What is the baseline that is used to correct the values to obtain amplitudes? If it is the whole period before the maximal RR value or the laser onset, wouldn't baseline values differ significantly across conditions and so potentially account for differences seen between conditions in the reported HR decelerations? Larger HR decelerations may be seen in conditions with higher HR simply as a regression to the mean phenomenon.

We thank the reviewer for this feedback and agree that additional clarification of Figure 3f–j is warranted.

For Figure 3f, the central line represents the peak RR value (maximal heart-rate deceleration) identified within the 2–7 s window following laser onset, rather than the laser onset itself. We realize this was not sufficiently clear and have revised the figure and corresponding Results text to clarify this point.

Regarding baseline correction, RR amplitudes were calculated as the difference between the RR at peak deceleration and the mean RR during the 8–10 s period preceding the RR peak, as described in the manuscript. Thus, the baseline was defined locally for each event and was not based on the entire pre-laser period or stimulation onset. We chose this approach to account for shifts in baseline heart rate across conditions and to capture the relative magnitude of the deceleration response rather than absolute RR values.

We appreciate the reviewer’s point regarding potential regression-to-the-mean effects, particularly in conditions with higher baseline heart rates. This is an important consideration. However, because the amplitude measure was baseline-corrected on an event-by-event basis, we believe the reported differences are unlikely to be explained solely by higher pre-stimulation heart rate. At the same time, our findings clearly show that elevated baseline heart rate influence the dynamic range of deceleration responses. Our findings show that under physiological conditions with elevated HR, larger heart-rate fluctuations would also contribute to increased HRV, which is often interpreted positively, despite potentially reflecting fragmented or dysregulated sleep states in this context.

To improve readability, we have revised the figure and associated text.

(Results, page 10). “To quantify the HR decelerations that happened after LC activation, we took the maximal RR value (so slowest HR) 2-7 s after LC stimulation and baseline corrected the value to the mean RR during the 8–10 s period preceding the RR peak to obtain their amplitude.”

(5) The findings regarding LC suppression could be further clarified.

(5A) Page 8: "observed a response in NE decline" - please be more precise. Did NE decline more or less?

We thank the reviewer for this suggestion and agree that the original wording was imprecise. To clarify the direction and nature of the response, we have revised the text to state:

(Results, page 11). “...we observed a gradual NE decline sustained throughout the laser period that was not observed in the YFP condition…”

(5B) It would be helpful to also show the correlation between NE and RR in the control (YFP) condition and whether there were any differences between YFP and Arch conditions (Figure 4e).

We thank the reviewer for this suggestion. We have now added the corresponding YFP correlation to Figure 4e. In the YFP group, the relationship between NE and RR showed a similar negative trend but did not reach statistical significance (*p* = 0.053). To directly assess whether the NE–RR relationship differed between Arch and YFP animals, we performed both a linear mixed-effects analysis and a Fisher r-to-z comparison.

Neither analysis revealed a significant difference between groups. The linear mixed-effects model showed no significant Group × NE interaction (slope difference = 0.994, *p* = 0.51), indicating that the NE–RR coupling was not altered by LC suppression. Similarly, Fisher's r-to-z comparison found no significant difference between the correlations (*p* = 0.42).

We believe this result is consistent with the relatively modest nature of the LC suppression paradigm. While Arch stimulation produced a clear reduction in NE levels, it did not induce a large shift in the overall NE–RR relationship. Instead, the data suggest that heart-rate responses remain coupled to noradrenergic fluctuations under both physiological conditions and during mild LC suppression. We have added the YFP data and clarified this interpretation in the revised manuscript.

(Results, page 12): “A similar negative relationship was observed in YFP controls (Fig. 4e), and the strength of the NE–RR association did not differ significantly between Arch and YFP animals (Suppl. File, Statistics), suggesting that LC suppression did not substantially alter the underlying coupling between these measures.”

(5C) This sentence took me multiple readings to understand - it would be helpful to rewrite to make it clearer: "indicating that, while HR generally did not respond strongly to LC suppression, the variability in RR responses was dependent on NE changes to the suppression (Figure 4e)."

We agree with the reviewer that this phrasing is hard to understand and we have optimized for better clarity. Please see new version below:

(Results, page 11/12). “Notably, despite the absence of a robust group-level HR effect, NE and RR responses remained negatively correlated across trials (Fig. 4e), indicating that HR dynamics continued to track the magnitude of noradrenergic suppression at the individual-response level”.

(5D) The two colors in Figure 4 are similar and hard to distinguish.

We agree that the colors are hard to separate and have altered them to make them easier to separate.

(5E) The correlations shown in Figure 4j seem to be driven by just two of the cases. Are the effects significant when outliers are removed?

We thank the reviewer for raising this point. To assess whether the observed correlation in Figure 4j was disproportionately driven by a small number of data points, we performed a formal outlier analysis using the ROUT method (Q = 1%). This analysis did not identify any statistical outliers in the dataset. Therefore, we did not have an objective basis for excluding any observations from the analysis.

(5F) Page 10: Were there any differences in memory performance between the Arch and YFP conditions?

We thank the reviewer for this question. The memory experiments were based on a previously published dataset (Kjaerby, Andersen et al., 2022), in which the primary objective was to assess the effect of LC suppression on sleep spindle dynamics and memory consolidation. In the present study, we performed an additional analysis by extracting heart-rate (RR) measures from these recordings to evaluate whether cardiac responses could serve as a biomarker of LC-mediated noradrenergic regulation.

However, due to technical limitations (EMG recording often suffers from noise) in extracting reliable RR signals from all animals in this dataset, the number of subjects available for this secondary analysis was reduced. All these considerations are described in Methods/Mice. As a result, we were not sufficiently powered to perform a direct statistical comparison of memory performance between Arch and YFP groups based on RR measures alone. Instead, we examined whether RR responses to LC suppression predicted behavioral performance across animals. When pooling Arch and YFP conditions, we observed a correlation between the magnitude of the RR response and subsequent memory performance, suggesting that heart-rate dynamics reflect noradrenergic modulation relevant for memory consolidation.

To avoid overinterpretation, we have therefore limited our conclusions to reporting this association rather than making direct group-level comparisons between Arch and YFP animals, and we have clarified this point in the revised manuscript.

(Results, page 13): “Interestingly, across pooled Arch and YFP animals, larger RR increases following LC suppression were associated with better subsequent memory performance. Additionally, RR and NE responses to LC suppression were negatively correlated indicating that animals showing stronger NE reductions also exhibited larger RR changes. Together, these findings suggest that heart-rate dynamics covary with noradrenergic responses during sleep and may reflect physiological processes relevant for sleep-dependent memory consolidation.”

(5G) Page 10: "We found a correlation between RR responses to LC suppression and sigma power, suggesting that a stronger HR reduction response is linked to higher spindle power." It should be noted in the text that the correlation was not specific to sigma (it was also seen for theta and beta, Figure 4i).

We agree with reviewer that this should be highlighted. We have changed the sentence:

(Results, page 12). “Furthermore, we found a correlation between RR responses to LC suppression and sigma power, suggesting that a stronger HR reduction response is linked to higher spindle power; similar correlations were also observed in the theta and beta frequency ranges (Fig. 4h-i).”

(6) It is not clear which of the sigma power and RR interval findings do/do not exactly line up between the mice and humans. It could be helpful to have a table comparing them. For instance, was the finding in humans that pre-HRB sigma power was positively associated with slowing in heart rate after the HRB also seen in mice? Was there evidence in mice (as seen in the human sample) that sleep-dependent memory improvement was associated with pre-HRB sigma power?

We thank the reviewer for this thoughtful comment and agree that the cross-species comparisons could be communicated more clearly. Our intention was not to imply exact one-to-one correspondence between all mouse and human findings, but rather to examine whether central–autonomic coupling surrounding phasic heart-rate events shows conserved features across species while acknowledging species-specific physiological differences.

Importantly, the mouse and human analyses were designed to address related but not identical questions. In mice, we leveraged optogenetic LC suppression to probe a more causal relationship between noradrenergic activity, heart-rate slowing, spindle-related dynamics, and memory consolidation. Previous work using this dataset demonstrated that 2-minute LC suppression robustly enhances spindle density and that spindle enhancement correlates with improved memory performance. In the present study, we therefore asked whether heart-rate slowing covaries with this LC-mediated spindle/memory relationship, supporting HR as a potential biomarker of these restorative processes.

By contrast, causal manipulation of LC activity is not feasible in humans. Instead, we focused on naturally occurring HRBs as putative downstream signatures of phasic LC– NE activity, motivated by our mouse findings that NE increases precede HR accelerations. We observed conserved coupling between sigma activity and HR dynamics across species, although the temporal profile differed, with sigma activity occurring closer to the HRB in humans than in mice. These temporal differences may reflect species-specific differences in cardiac and sleep physiology.

Regarding the reviewer’s specific questions, the positive relationship between pre-HRB sigma power and post-HRB heart-rate slowing was tested in humans, where this metric showed the strongest relationship to behavioral outcome. We did not directly test the same measure in mice because, unlike humans, HR recovery following HRBs did not show a pronounced baseline shift (Fig. 5b), limiting the interpretability of this comparison. Similarly, we did not directly correlate pre-HRB sigma power with memory performance in mice, as the more causal LC suppression paradigm already demonstrated a spindle–memory relationship in this species and was the focus of our mechanistic analysis.

To reduce confusion, we have revised the Results conclusion to make a clearer overview:

(Results, page 14): “In conclusion, mice and humans displayed evidence of conserved autonomic-central coupling, reflected in coordinated HR and sigma power dynamics surrounding phasic cardiac events.

However, the temporal relationship between these events differed across species, likely reflecting differences in sleep and cardiovascular physiology. In mice, causal manipulation of LC activity demonstrated that heart-rate dynamics covary with LC-mediated noradrenergic and spindle-related processes linked to memory consolidation. In humans, sigma power preceding HR bursts was associated with both post-HRB heart-rate slowing and sleep-dependent memory improvement, suggesting that autonomic–central coupling surrounding HR events may provide a translational marker of restorative sleep processes (Fig. 5n).”

(7) Page 18: It is not clear if the sex of mice was balanced across controls and optogenetics groups.

We thank the reviewer for this important comment and agree that the sex distribution should be reported more clearly. These experiments relied on the availability of animals from the heterozygous TH-Cre transgenic line, and given the relatively small cohort sizes, perfect balancing across sex and experimental groups was not always feasible.

For the LC activation experiments, the sex distribution was: YFP: 2 male / 2 female; ChR2: 4 male / 2 female. For the LC suppression experiments, the distribution was: YFP: 4 female; Arch: 3 female / 1 male.

Although the groups were not perfectly sex balanced, we had no strong reason to expect robust sex-dependent differences in the physiological effects of these optogenetic manipulations, particularly given the relatively strong and acute nature of the intervention. At the same time, we acknowledge that the present study was not powered to assess sex as a biological variable, and subtle sex-dependent effects therefore cannot be excluded. To improve transparency, we have now clarified the sex distribution in the Methods/Mice section.

**Reviewer #2 (Public review):**

Summary:

The major part of this study reproduces previously published findings in both mice and humans and provides incremental analyses on these findings. In essence, the work reaffirms the presence of coordinated infraslow fluctuations in sigma power and heart rate during NREM sleep. It further confirms previous findings that coordination depends on noradrenaline-releasing neurons in the locus coeruleus. Also supporting previously published work in mice and humans, the authors describe a link between the strength of these infraslow fluctuations and memory consolidation in mice and humans.

Strengths:

The authors successfully replicate key previously reported phenomena across both mice and humans. Confirmatory studies and demonstrations of reproducibility are essential for progress in neuroscience. To maximize their value, such studies should clearly acknowledge their confirmatory nature and carefully situate what, in their view, are novel results, going beyond existing literature.

Weaknesses:

The authors' interpretation of their data needs to be revised. Many of their claims regarding the mechanistic basis of their findings and the predictive value of their correlative datasets are not supported by the available evidence.

In the present manuscript, several citations of literature on the work they reproduce lack precision or completeness, which reduces transparency and obscures how the reported findings relate to previously established results.

We thank Reviewer 2 for the thoughtful comment regarding positioning of our findings relative to the literature, and caution in mechanistic interpretation. In response, we have revised the Introduction, Results, and Discussion to more clearly acknowledge foundational studies in this area and to better clarify how the present work extends beyond them.

We agree that prior work has demonstrated infraslow coupling between sigma activity, norepinephrine (NE) dynamics, and heart rate (HR), and has established a role for the locus coeruleus (LC) in coordinating these oscillations. However, cardiac measures in these studies were typically treated as secondary observations rather than as primary experimental targets. A central goal of the present study was therefore to provide a systematic and mechanistically grounded characterization of NE-mediated HR dynamics during sleep across multiple timescales, including infraslow oscillations, sleep–wake transitions, and causal manipulations of LC activity.

Importantly, we also aimed to relate infraslow HR fluctuations to the very-low-frequency (VLF) component of heart rate variability (HRV), which remains comparatively under-characterized and mechanistically unresolved in the clinical HRV literature. By linking LC activity, NE dynamics, and HR fluctuations across behavioral states, our findings provide a biologically grounded framework that may help explain this component of HRV.

A second major objective of the study was translational. Because direct LC recordings are not feasible in humans, we asked whether cardiac dynamics alone could reflect the infraslow, memory-consolidating potential of sleep and thus serve as a noninvasive biomarker. By directly manipulating LC activity and demonstrating corresponding changes in HR dynamics, our results strengthen the mechanistic rationale for using HRV—particularly its VLF component—as an accessible proxy of LC-dependent sleep physiology.

We therefore respectfully disagree with the suggestion that the present study does not provide novel insight. Rather, the revised manuscript now more clearly emphasizes that our contribution lies in (i) systematically characterizing NE-dependent HR dynamics across sleep states, (ii) linking these dynamics to the poorly understood VLF component of HRV, and (iii) establishing a causal and translational framework for using cardiac measures as markers of LC-mediated sleep processes.

We hope the reviewer finds that the revised Introduction and Discussion better highlight both the existing literature and the specific advances provided by the present work.

**Recommendations for the authors:**

**Reviewer #1 (Recommendations for the authors):**

I have been convinced by discussions about the replicability crisis that it should be a standard practice to share data upon publication in a publicly accessible online repository such as Open Science Framework or OpenNEURO. Making data available can increase the impact of the research study. Simply stating "data available upon request" as done in the current draft is not sufficient as, unfortunately, when data are not shared upon publication in a public repository, it can be impossible to gain access by request from the researchers - the majority of requests from other researchers to obtain data are not complied with (e.g., Vanpaemel, Vermorgen, Deriemaecker, & Storms, 2015; Wicherts, Bakker, & Molenaar, 2011).

We fully agree with the reviewer about the importance of data sharing for transparency, reproducibility, and maximizing the impact of research. Consistent with these principles, we will make the human dataset publicly available on the Open Science Framework upon publication and have added the link to this repository in the manuscript under Data Availability (https://osf.io/g6emj/). With respect to the mouse dataset, we respectfully note that this dataset is currently the subject of multiple planned and ongoing analyses that extend beyond the scope of the present manuscript. Releasing these data publicly at this stage could compromise these efforts and lead to potential misinterpretation prior to completion of the full analytic pipeline. For this reason, we believe that it would not be appropriate to share the mouse data in a public repository at this time. However, we remain committed to transparency and will make the mouse data available upon reasonable request during this period, with the intention of publicly releasing the dataset once the planned analyses are complete.

(1) p. 4: There is some orphan text at the top of the page ("marker of Alzheimer's disease. Furthermore, maintaining LC neural density prevents neurodegeneration (13). Given the reported reduction in HRV in aging and Alzheimer's disease, suppressed").

Thank you. We have removed the orphan text.

(2) p. 22: What does NFR refer to?

Novel-to-familiar ratio. We have removed the abbreviation from the main text. It is now only used in the figure.

(3) I might have missed this information, but it was not clear to me how the epochs to be analyzed were selected, and for the mice, how much wake vs. sleep they included.

We thank the reviewer for this comment and apologize that the epoch selection criteria were not sufficiently clear. They were mentioned under Methods. For the optogenetic analyses in mice, epochs were selected based on NREM sleep including microarousals to ensure that physiological responses were evaluated within stable sleep conditions while allowing for natural brief interruptions.

For the LC activation (ChR2) experiments, stimulation epochs were included only if NREMinclMA began at least 30 s before laser onset and continued for at least 30 s after laser onset. For the LC suppression (Arch) experiments, the criterion was similarly ≥30 s of NREMinclMA prior to laser onset, but extending ≥60 s following laser onset to accommodate the longer suppression response profile.

Thus, analyses were intentionally restricted to sleep periods, and wakefulness was not included except where it emerged naturally as an outcome of the manipulation or transition under investigation. We have now clarified these inclusion criteria in the Methods/ Event marker selection section to improve transparency.

(4) In the Supplement, Figure 3 appears before Figure 2.

Thanks. We have corrected it.

Signed, Mara Mather

**Reviewer #2 (Recommendations for the authors):**

The authors' interpretation of their data needs to be revised. Many of their claims regarding the mechanistic basis of their findings and the predictive value of their correlative datasets are not supported by the available evidence.

There are three major directions in which this study would need to be revised.

Part 1 - Literature citations of both mouse and human literature need to be revised, and citations placed in a manner that accurately reflects what has been previously done. In detail:

(1.1) The statement on p. 4 regarding "the extent to which phasic infraslow NE fluctuations ...is not well understood" disregards a previous publication in which closed-loop optogenetic stimulation of LC was already shown to regulate HR variations on the infraslow time scale (10.1016/j.cub.2021.09.041).

We thank the reviewer for this important point. The study by Osorio-Forero et al. (2021) was already cited as ref. 4 in our original manuscript and in the discussion, we specifically highlighted this study: ‘These findings build on Osorio-Forero et al. (35) as well as other studies’; however, we agree that our wording did not sufficiently emphasize its key finding that closed-loop optogenetic manipulation of LC activity can coordinate infraslow heart-rate fluctuations and spindle clustering during NREM sleep.

We have revised the relevant paragraph in the introduction to explicitly acknowledge that ref. 4 demonstrated causal coordination between LC activity, sleep spindle dynamics, and heart-rate fluctuations. We have also refined our statement of the knowledge gap to clarify which novel questions our study addresses. We believe these revisions more accurately position our work within the existing literature and clearly distinguish our contributions from prior studies.

We have updated the introduction in several places to address these comments:

(Introduction, page 4). “It has previously been reported that HR fluctuates at similar infraslow frequencies as NE fluctuations and sigma power in mice (Lecci et al., 2017; Osorio-Forero et al., 2021) and that phasic HR fluctuations correlate with infraslow changes in pupil diameter (Carro-Domínguez et al., 2025), a proxy for changes in NE levels (Murphy et al., 2014; Reimer et al., 2016). Importantly, optogenetic manipulation of the LC has demonstrated that infraslow LC activity coordinates sleep spindle clustering and heart rate fluctuations during NREM sleep (Osorio-Forero et al., 2021). Together, these findings support a functional coupling between the central LC–NE system and peripheral cardiac dynamics, further supported by findings that HR increases accompany MAs during NREM sleep (Carro-Domínguez et al., 2025; Osorio-Forero et al., 2025).”

(Introduction, page 4/5). “Due to the frequency overlap with VLF HRV, we wondered if central infraslow NE dynamics could be linked to this poorly understood HRV indicator. Furthermore, are infraslow NE fluctuations directly reflected by heart-rate variability under different physiological states or does LC–HR coupling scales differently with LC output? Specifically, if infraslow NE oscillations display faster frequencies - as occurs during sleep fragmentation - will cardiac dynamics exhibit corresponding changes? Conversely, given that stronger infraslow NE dynamics correlate with memory consolidation through their regulation of sleep spindles, could the peripheral autonomic signatures provide an accessible cross-species biomarker of spindle-dependent memory consolidation? Addressing these questions could help bridge mechanistic insights into LC-mediated sleep regulation with established HRV metrics used in human physiology.”

(1.2) In this same published paper, optogenetic stimulation of LC was already used to "determine the causal relationship..." (p.6). However, the authors do not cite these data.

We thank the reviewer for this comment. As noted in our response to Comment 1.1, ref. 4 was already cited in the Introduction, and we have now revised that section to more explicitly emphasize this paper. In addition, we have modified the wording in the Results section.

(Results, page 8). “After finding the inverse correlation between NE and RR in natural sleep transitions, we next sought to further characterize the causal influence of LC activity on HR dynamics, using a closed-loop optogenetic approach to modulate NE oscillatory frequency during NREM sleep.”

(1.3) The authors' speculation about LC-induced sympathetic and parasympathetic actions is premature: this study does not provide pharmacological experiments in this direction. However, two published studies implied a parasympathetic mechanism linked to infraslow fluctuations of LC activity (10.1016/j.cub.2021.09.041, 10.1016/j.cub.2017.12.049). These findings should be appropriately cited. While HR decelerations may reflect compensatory autonomic responses, there is no direct evidence in the present study that these effects are sympathetically mediated. An alternative, and equally plausible, interpretation is enhanced parasympathetic activity. This distinction is particularly important given that the observed increases in mean HR during LC stimulation cannot distinguish between reduced parasympathetic activity and increased sympathetic drive.

We thank the reviewer for raising this important point and agree that the current study does not provide direct mechanistic evidence to disentangle sympathetic versus parasympathetic contributions to LC-mediated heart rate regulation. This was not the intention of our study; rather, the relevant discussion section was meant to provide mechanistic interpretations and hypotheses based on the observed physiology. To avoid overstating our conclusions, we have revised the wording to more clearly emphasize the speculative nature of these interpretations. Furthermore, we have added the suggested references demonstrating that muscarinic blockade reduces heart rate and pupil fluctuations during sleep, which support the possibility of a parasympathetic contribution to infraslow LC-related dynamics.

(Discussion, page 16/17). “Prior findings demonstrate that LC is linked to the autonomic nervous system. Stimulation of LC projections decrease parasympathetic cardiac vagal activity (Wang et al., 2014) and also influences sympathetic output through direct projections to the preganglionic cells in the sympathetic nervous system (Karemaker, 2017; Nygren and Olson, 1977; Samuels and Szabadi, 2008). While the mechanisms generating VLF HRV are not well defined (Armour, 2003; Shaffer et al., 2014; Wang et al., 2014), there is a clear parasympathetic component (Taylor et al., 1998). This combined with the ability of pharmacological blockage of the parasympathetic system to block infraslow oscillations of HR (Osorio-Forero et al., 2021) and pupil diameter during sleep (Yüzgeç et al., 2018), led us to expect a slowing of HR during LC suppression due to parasympathetic disinhibition.”

(1.4) It is not clear why prefrontal NE signals were associated with HR fluctuations. Literature evidence indicates other brain areas that are functionally more directly linked to autonomous fluctuations.

We thank the reviewer for raising this point. We do not speculate that mPFC NE activity is causally linked to heart rate fluctuations or that the mPFC directly mediates the observed autonomic dynamics. Instead, mPFC NE signaling was used as an experimentally accessible readout of LC activity. Importantly, accumulating evidence suggests that infraslow NE oscillations are globally coordinated phenomena that are expressed across multiple brain regions during sleep, making mPFC NE a valid proxy for LC-driven neuromodulatory state dynamics. We have clarified this in the result section:

(Results, page 6). “mPFC was selected as a cortical readout of LC-mediated norepinephrine dynamics, as infraslow NE fluctuations are coordinated across widespread brain regions.”

(1.5.a) The lack of effect of Arch-inhibition of LC on infraslow NE signals is concerning. Prior work showed that bilateral LC inhibition does affect NE signals and also infraslow sigma power fluctuations (10.1016/j.cub.2021.09.041, 10.1038/s41593-024-01822-0). This discrepancy should be explicitly acknowledged and discussed on p.9.

We thank the reviewer for highlighting this important point. To clarify, we did observe a robust effect of LC inhibition on noradrenergic signaling, with clear suppression of the NE signal following Arch-mediated LC inhibition (Fig. 4c), aligned to laser onset. In addition, LC suppression increased neuronal synchronization, including enhanced sigma power relative to YFP controls (Fig. 4h), consistent with previous reports showing that reduced LC activity promotes synchronized sleep-related oscillations. Thus, we do not interpret our findings as indicating an absence of LC suppression effects.

The apparent discrepancy relates specifically to the absence of a statistically significant group-level shift in infraslow NE or HRV power during NREM sleep, rather than the efficacy of the manipulation itself. We note that our inhibition paradigm was intentionally mild, consisting of repeated 2-minute suppression periods separated by 4-minute intervals, and was designed to introduce subtle shifts within physiological ranges rather than globally reorganize infraslow sleep structure. Accordingly, we consider the immediate NE and heart-rate responses to LC inhibition to be the most sensitive physiological readouts of LC-mediated regulation in this context.

We also note that the studies cited by the reviewer used different suppression paradigms, including more frequent manipulations. Thus, while our suppression scheme did not result in detectable infraslow reorganization, we do not believe they reflect an ineffective LC suppression as we demonstrated a clear NE reduction in response to time-locked LC suppression.

We have added a sentence to the result section to explain the lack of effect infraslow power:

(Results, page 12). “This likely reflects the relatively mild and intermittent LC suppression paradigm, which was designed to remain within physiological ranges and therefore did not globally reorganize infraslow sleep dynamics.”

(1.5.b) Additionally, the authors state in the Discussion that they "find no consistent modulation in HR during LC suppression, suggesting that the LC-HR connection is more strongly associated with sympathetic activity rather than parasympathetic inhibition". However, the results primarily demonstrate an absence of modulation in mean HR, while preserving a significant relationship between RR intervals and stimulation. This indicates a modulation of heart rate variability, even in the absence of changes in average HR. Notably, such variability-related effects may fall outside the VLF range and could instead involve higher-frequency components.

We thank the reviewer for this important clarification. We agree that our original wording may have conflated the absence of modulation in mean HR with the absence of autonomic modulation more generally. To address this point, we revised the Discussion to emphasize that LC activity may influence VLF HRV through sympathetic activation and/or indirect modulation of cardiac vagal activity, even in the absence of robust changes in average HR. We have softened our previous interpretation that the LC-HR relationship is primarily sympathetic in nature and instead discuss a more nuanced interaction between sympathetic and parasympathetic influences on HRV dynamics.

(Discussion, page 16/17). “Prior findings demonstrate that LC is linked to the autonomic nervous system. Stimulation of LC projections decrease parasympathetic cardiac vagal activity (Wang et al., 2014) direct projections to the preganglionic cells in the sympathetic nervous system (Karemaker, 2017; Nygren and Olson, 1977; Samuels and Szabadi, 2008). While the mechanisms generating VLF HRV are not well defined (Armour, 2003; Shaffer et al., 2014; Wang et al., 2014) there is a clear parasympathetic component (Taylor et al., 1998). This combined with the ability of pharmacological blockage of the parasympathetic system to block infraslow oscillations of HR (Osorio-Forero et al., 2021) and pupil diameter during sleep (Yüzgeç et al., 2018) led us to expect a slowing of HR during LC suppression due to parasympathetic disinhibition. Interestingly, we found no consistent modulation in HR during LC suppression, suggesting that the LC-HR connection may also somehow be driven by sympathetic outflow. Previous research had indicated that LF power may also represent sympathetic activity, but this interpretation has been challenged due to the mixed contribution of both autonomic branches (Houle and Billman, 1999; Japundzic et al., 1990; Reyes et al., 2013). LF and HF ratio (LF/HF) were traditionally thought to reflect balance between sympathetic and parasympathetic activities (i.e., the sympatho-vagal balance), though currently considered as an oversimplification of non-linear integration of autonomic signals (Billman, 2013; Pagani et al., 1986). Our findings implicate the VLF may offer a precise marker for central arousal states, given its overlaps with infraslow phasic fluctuations of LC-NE levels. Although parasympathetic activity appears important for the expression of VLF oscillations, growing evidence suggests that VLF dynamics reflect broader interactions between the heart and autonomic nervous system rather than simple sympathetic or parasympathetic control alone (Armour, 2003; Shaffer et al., 2014). Within this framework, infraslow LC–NE dynamics may represent one central contributor to these slow cardiac fluctuations during sleep either directly through sympathetic activation or indirectly by inhibition of cardiac vagal activity.”

(1.6) The relationship between sigma power fluctuations and HR is different in humans than in mice. This has been shown before (10.1126/sciadv.1602026, 10.1038/s41593025-02159-y). This work should be mentioned on p. 11.

We already acknowledge prior studies demonstrating species differences in the relationship between sigma power fluctuations and heart rate in the discussion section. However, to accommodate the reviewer’s comment and improve clarity for the reader, we have now also added the suggested references to the Results section, where the relationship between sigma power fluctuations and HR is first discussed.

(Results, page 14). “These temporal differences may reflect species-specific physiology differences in cardiac timescales, which has also been previously reported (Bergel et al., 2025; Carro-Domínguez et al., 2025; Lecci et al., 2017).”

(1.7) Correlations between the strength of infraslow sigma power fluctuations and memory consolidation have been published and should be discussed (10.1126/sciadv.1602026). It is surprising to see that correlations with learning in humans are done using pre-HRB sigma peaks rather than heart rate. This is a measure that is very close to the one used by Lecci et al.; this similarity should be clearly acknowledged. The way the data are currently presented limits this manuscript's novelty, also in its translational aspect.

We agree that the work by Lecci et al. (2017) established an important relationship between the association of infraslow sigma power fluctuations and memory consolidation, which is highly relevant to our findings.

Importantly, the underlying infraslow fluctuations in neuromodulatory tone are increasingly recognized as key regulators of sleep spindle dynamics (sigma power), including from our own previous work demonstrating that direct manipulation of locus coeruleus–norepinephrine infraslow rhythms alters spindle organization and sleep continuity. Thus, our findings are conceptually aligned with prior studies linking sigma fluctuations to memory consolidation.

However, we would like to clarify an important distinction in our translational approach. While Figure 4 demonstrates that heart rate dynamics during sleep can predict memory performance in mice, Figure 5 was designed to address the translational potential of these findings in humans, where direct neuromodulatory readouts are not readily accessible. Here, we deliberately focused on heart rate bursts and their associated sleep dynamics as a clinically tractable physiological measure.

We acknowledge that the pre-HRB sigma increase may appear conceptually similar to the measure used by Lecci et al.; however, our approach is not equivalent. Rather than selecting spindle or sigma peaks themselves, we aligned analyses to heart rate accelerations and examined the robust upregulation of sigma activity preceding these events. In this framework, sigma activity serves as a physiological readout linked to autonomic dynamics, rather than being the primary anchor of analysis. We chose this measure because heart rate bursts are influenced by multiple physiological factors, and the associated sigma dynamics provided the clearest and most robust relationship with memory outcomes in the human dataset.

We have revised the Discussion to more clearly acknowledge the similarity to prior work. We believe our findings extend prior observations by providing evidence that sleep-related heart rate fluctuations may serve as a non-invasive readout of the memory-preserving function of sleep.

(Discussion, page 18). “Previous work in humans demonstrated that the strength of infraslow sigma oscillations correlates with sleep-dependent memory consolidation in humans (Lecci et al., 2017).”

(1.8) Conclusions as to whether sigma fluctuations might be slightly slower and less powerful in mice are not justified. More work is required to determine which infraslow manifestations are most useful for cross-species comparisons. Moreover, little is currently known about LC activity in human sleep. A careful look into how pupil diameter correlates with sigma power should provide clues for further discussion (see Carro-Dominguez et al). A detailed study of infraslow fluctuations in human sleep should also be discussed https://doi.org/10.1101/2024.11.06.620875.

We thank the reviewer for this thoughtful comment. We agree that our original phrasing suggesting that sigma dynamics in mice may be “slower and less powerful” than in humans was overly interpretive. We have removed this sentence from the Results section. In addition, we have expanded the Discussion to more thoroughly integrate recent human literature on infraslow sleep dynamics.

(Discussion, page 19). “Importantly, infraslow fluctuations of sigma power in human sleep have received growing attention. Recent work demonstrates that the infraslow fluctuation of sigma power segments N2 sleep into functional phases associated with arousal and memory-related sleep markers (Dimitriades et al., 2024). Complementary findings using pupillometry show that pupil diameter fluctuates on similar infraslow timescales during NREM sleep and is inversely related to spindle clustering, providing indirect evidence that arousal-related noradrenergic dynamics shape human sleep microstructure (Carro-Domínguez et al., 2025). However, LC activity during human sleep remains inferred rather than directly measured, and systematic perturbation studies linking LC output to spindle–autonomic coupling in humans are currently lacking. Together, these observations underscore both the promise and the current limitations of cross-species comparisons of infraslow sleep dynamics.”

(1.9) Citation of literature should be as explicit as possible. Referring to "many studies rely on plasma levels..." while including some that actually did real-time fiber photometric measures is misleading.

We thank the reviewer for this suggestion and agree with the reviewer about the importance of accurately representing prior studies. We have now updated the discussion to clarify this.

(Discussion, page 15). “Many studies have linked HR to NE (Fawaz and Simaan, 1963; Sundaram et al., 1991; Tanoue et al., 2022; Watson et al., 1979). Many rely on plasma levels of NE, which, while linked to central NE (Gurguis and Uhde, 1998), has low temporal resolution, making causal interpretations harder. In recent years, the use of biosensors and fibre photometry allows for very reliable estimate of the temporal dynamics of NE changes making association to HR more precise (Osorio-Forero et al., 2021).”

(1.10) Regarding the discussion on the baroreflex: The emphasis is placed predominantly on sympathetically mediated effects. However, the description of the baroreflex loop is incomplete, as it overlooks the substantial contribution of parasympathetic modulation. In particular, heart rate adjustments within the baroreflex are primarily mediated by parasympathetic mechanisms.

We agree with reviewer that this important notion should be added. We have rephrased the discussion as below:

(Discussion, page 20). “These neurons suppress the activity of the rostral ventrolateral medulla, ultimately resulting in reflex parasympathetic activation with sympathetic inhibition lowering the HR (Aicher et al., 2000; Lanfranchi and Somers, 2002).”

(1.11) Regarding the interpretation of HRV analysis, the discussion places disproportionate weight on sympathetic modulation in the interpretation of HRV metrics. This framing is inconsistent with recent conceptual clarifications, including a recent Nature Reviews Cardiology article by Menuet et al. (10.1038/s41569-02501160-z), which cautions against simplistic low-frequency/high-frequency (LF/HF) interpretations of autonomic balance.

We thank the reviewer for this thoughtful comment. We agree that HRV frequency bands should not be interpreted as exclusive markers of specific autonomic branches. In the original manuscript, we cited Billman (2013), which challenges the validity of LF/HF as a measure of sympatho-vagal balance, to acknowledge these conceptual limitations. However, we recognize that some of our phrasing, particularly in the section discussing compensatory HR decelerations, may have implied branch-specific dominance.

We have updated the Discussion section as follows:

(Discussion, page 17). “Interestingly, we found no consistent modulation in HR during LC suppression, suggesting that the LC-HR connection may also somehow be driven by sympathetic outflow. Previous research had indicated that LF power may also represent sympathetic activity, but this interpretation has been challenged due to the mixed contribution of both autonomic branches (Houle and Billman, 1999; Japundzic et al., 1990; Reyes et al., 2013). LF and HF ratio (LF/HF) were traditionally thought to reflect balance between sympathetic and parasympathetic activities (i.e., the sympatho-vagal balance), though currently considered as an oversimplification of nonlinear integration of autonomic signals (Billman, 2013; Pagani et al., 1986). Our findings implicate the VLF may offer a precise marker for central arousal states, given its overlaps with infraslow phasic fluctuations of LC-NE levels. Although parasympathetic activity appears important for the expression of VLF oscillations, growing evidence suggests that VLF dynamics reflect broader interactions between the heart and autonomic nervous system rather than simple sympathetic or parasympathetic control alone (Armour, 2003; Shaffer et al., 2014). Within this framework, infraslow LC–NE dynamics may represent one central contributor to these slow cardiac fluctuations during sleep either directly through sympathetic activation or indirectly by inhibition of cardiac vagal activity.”

We have also replaced the title of the Discussion section title “Locus-coeruleus-mediated sympathetic control drives compensatory heart rate decelerations” with “Locus-coeruleus activation drives compensatory heart rate decelerations via autonomic feedback mechanisms”

(1.12) In particular, the manuscript attributes VLF power primarily to sympathetic activity, despite evidence that very-low-frequency RR-interval oscillations are strongly dependent on parasympathetic integrity. Notably, parasympathetic blockade has been shown to nearly abolish VLF oscillations in humans (Taylor et al., Circulation, 1998; doi:10.1161/01.CIR.98.6.547).

We thank the reviewer for highlighting this important point. It was not our intention to imply that VLF HRV is driven primarily by sympathetic activity or to disregard the important role of parasympathetic integrity in shaping VLF oscillations. Our intention was to present the multifactorial and incompletely resolved nature of the VLF component; however, if our wording can be interpreted otherwise, we agree that clarification is warranted.

In response, we have revised the manuscript to better reflect the current understanding of VLF physiology. Specifically, we now make clearer that, while VLF HRV remains less mechanistically defined than HF and LF HRV, substantial evidence supports a strong parasympathetic contribution to the expression of VLF oscillations. We have incorporated the reviewer-suggested references within this comment and others and clarified this point throughout the revised manuscript.

(Discussion, page 16). “While the mechanisms generating VLF HRV are not well defined (Armour, 2003; Shaffer et al., 2014; Wang et al., 2014) there is a clear parasympathetic component (Taylor et al., 1998).”

(Discussion, page 17). “Although parasympathetic activity appears important for the expression of VLF oscillations, growing evidence suggests that VLF dynamics reflect broader interactions between the heart and autonomic nervous system rather than simple sympathetic or parasympathetic control alone (Armour, 2003; Shaffer et al., 2014).”

(1.13) Moreover, based on work by Armour (2003) and Kember et al. (2000, 2001), the VLF rhythm is thought to emerge from stimulation of afferent sensory neurons within the heart, further arguing against a purely sympathetic interpretation. Together, these findings indicate that the discussion overemphasizes sympathetic mechanisms and underrepresents the contribution of parasympathetic and afferent cardiac pathways to HRV, particularly in the VLF range.

We thank the reviewer for this important point. Our response to this comment is largely aligned with our response to comment (1.12). In the revised Discussion, we now more clearly acknowledge that VLF oscillations likely arise from more complex cardioautonomic interactions than simple sympathetic and parasympathetic innervation alone. At the same time, we have intentionally avoided an extensive discussion of these mechanisms, as our experimental design does not directly address these pathways.

Part 2 - There are a number of conceptual issues that need more careful elaboration:

(2.1) A highly problematic point throughout this study is the choice of AUCs, notably of NE signals and RR intervals, rather than the signal amplitudes. It confuses the correlations to events of different durations, such as MAs and wakefulness. It is not possible to draw conclusions of the kind "cardiac rhythm are tightly coupled with the infraslow phasic NE ..." because such statements ignore that AUCs conflate amplitude and duration of a signal.

We thank the reviewer for this comment and agree that the distinction between amplitude- and duration-related signal features is important. We deliberately chose AUC measures because our intention was to capture the overall physiological response over time, including both the magnitude and temporal evolution of the signal, rather than relying solely on a single peak value. In this context, AUC provides information about the shape and sustained nature of NE and RR changes within a defined time window, which we considered particularly relevant for temporally dynamic responses.

At the same time, we appreciate the reviewer’s concern that AUC may conflate response amplitude and duration, particularly when comparing events of different lengths such as microarousals and wakefulness. To minimize this issue, the AUC windows were intentionally kept relatively short and fixed, thereby limiting the influence of prolonged wake episodes or differences in transition duration on the measure. Thus, our intention was not to quantify the total duration of awakenings, but rather the immediate physiological response profile surrounding the event.

To address this concern more directly, we compared amplitude- and AUC-based measures across all mice. The results are now shown in Suppl. Figure 1g. We found a strong correspondence between amplitude and AUC measurements for NE signals, indicating that the observed relationships are not dependent on the choice of metric. A similar, albeit weaker, relationship was observed for RR responses. This likely reflects physiological constraints on heart-rate dynamics, where the initial heart-rate acceleration is relatively similar across vigilance-state transitions (Figure 1e), while the duration of the response differs substantially. As a result, amplitude measures may underestimate differences between transitions, whereas AUC better captures the extent of the cardiac response. Importantly, direct comparison of NE and RR amplitudes still revealed a significant relationship, supporting the overall conclusion that cardiac and noradrenergic responses are coupled. However, this relationship was weaker than that observed using AUC measures, suggesting that incorporating temporal aspects of the response captures additional biologically relevant information.

In addition to the figure, we have revised the Results section to include these considerations:

(Results, page 7): “These comparisons were performed using area under the curve (AUC) estimates of NE and R-R responses. Importantly, comparing AUC and peak amplitude measures showed a similar overall relationship, and the coupling between NE and RR remained significant when only peak amplitudes were considered (Suppl. Fig. 1g), indicating that the findings are not solely driven by the response duration aspect of AUC. The somewhat stronger relationship observed with AUC-based measures may reflect rapid saturation of heart-rate responses across vigilance-state transitions, making response persistence an informative component of the physiological signal.”

(2.2) A next problematic aspect of the study is the analysis of noradrenergic signals and HR at transitions (e.g., from NREM sleep to sleep wakefulness or to microarousals). The abstract does not mention these data, leaving open how they fit into the paper's message. There are also two problems with it: (a) Noradrenaline levels increase with wakefulness, as do many other neuromodulators. This is not novel. Furthermore, why use an AUC measure for 0-25 s when MAs last only 5 or 15 s? (b) biosensor signal comparisons are difficult to make for state transitions, because blood flow changes and modifies the fluorescent signal.

We thank the reviewer for these comments and appreciate the opportunity to clarify the rationale and interpretation of these analyses.

Regarding the inclusion of vigilance-state transitions, our intention was not to claim novelty in the observation that NE levels increase during wakefulness. Rather, these analyses were included to provide an additional physiological context in which to examine the coupling between NE and heart-rate dynamics. Specifically, the transition analyses allowed us to determine whether graded changes in NE across sleep-to-wake transitions were mirrored by corresponding RR changes (Fig. 1d–e) and whether these responses covaried (Fig. 1f), thereby strengthening the overall conclusion that cardiac dynamics track noradrenergic signaling across naturally occurring sleep-state fluctuations.

Regarding the use of AUC measures, we deliberately chose this metric because it captures the overall physiological response over a defined time window, including both magnitude and temporal evolution, rather than relying solely on a peak value. In this context, AUC was intended to reflect differences in the overall response profile, for example that NE and RR responses during microarousals may return more rapidly toward baseline than during sustained wakefulness. To minimize the influence of differing event durations, the analysis window was kept fixed (0–25 s) across all transition types. Importantly, we directly compared AUC- and amplitude-based measures and found that they produced largely similar relationships (Suppl. Fig. 1g). The coupling between NE and RR responses remained significant when only peak amplitudes were considered, indicating that the observed relationship is not solely driven by response duration. However, the relationship was somewhat stronger with AUC-based measures, likely because heart-rate responses rapidly saturate across vigilance-state transitions, making response persistence an informative component of the physiological signal. As mentioned in the previous comment, we have added a Figure and new result text to highlight this.

Regarding the concern about blood-flow related artifacts in fluorescent biosensor signals, we agree that hemodynamic contamination is an important consideration for neuromodulator recordings and applies broadly to biosensor-based measurements, including analyses of infraslow fluctuations. To minimize this issue, ΔF/F calculations were performed using the isosbestic control channel, which serves to correct for movement- and hemodynamic-related signal fluctuations. Furthermore, hemodynamic artifacts typically occur rapidly at state transitions, whereas GRAB-NE signals display slower dynamics. If uncorrected hemodynamic contamination strongly influenced the signal, we would expect abrupt signal distortions tightly aligned to arousal onset, which was not evident in our recordings. While we cannot completely exclude residual hemodynamic influences, we do not believe they account for the graded NE responses observed across vigilance-state transitions or the corresponding relationship with RR dynamics.

(2.3) Wordings such as 'extent of arousal' to compare MAs and wakefulness are problematic. Microarousals and wakefulness are qualitatively different behaviorally, physiologically, and in terms of neuromodulatory conditions

We thank the reviewer for the helpful comment. Our intention was not to imply that microarousals and wakefulness are qualitatively identical states differing only in magnitude. Rather, we used arousal in the broader neurophysiological sense, referring to the degree of activation of central arousal systems, with wakefulness representing part of this continuum. However, we recognize that in the sleep field, arousal is often used more specifically to describe brief EEG desynchronization events and that we furthermore use micro-arousals as a broad term for these sleep arousals, which may make our wording confusing. To avoid ambiguity, we have revised the manuscript to replace this terminology with vigilance state, sleep–wake state, or state transitions, depending on the context.

(2.4) To interpret linear correlations between datasets, even for the ones with Rsquare values < 0.5, as 'predictive' represents an overextended interpretation that is not supported by available evidence. This concern is aggravated due to the use of AUCs that confound amplitudes and time courses. This is in particular the case for Figure 5d, 5k, or 5m…

We thank the reviewer for this important comment. We agree that the term predictive may overstate the interpretation of these correlations, particularly given the modest R² values in some analyses. Our intention was to highlight an association between heartrate and NE-related measures rather than imply strong predictive performance or causality. We have therefore revised the wording throughout the manuscript to avoid predictive language and instead refer to these relationships as associations or correlations.

Regarding the use of AUC, we selected summary metrics based on the physiological characteristics of the signal of interest. In cases where responses were characterized primarily by rapid shifts to a new level, amplitude measures were used. In contrast, AUC was chosen when both the magnitude and duration of the response were considered physiologically relevant.

Part 3. A substantial number of experimental and analytical points require clarification. Here is a list of a few examples; many observations noted here apply equally to other figure panels.

(3.1) Many figure panels leave it open about whether averages or representative data are shown, how many animals are included, and what kind of measures are plotted.

We thank the reviewer for this comment and agree that clarity in figure presentation is important. In response, we have carefully revised the figure legends throughout the manuscript to more explicitly state whether data shown are representative examples or group averages, clarify the number of animals included in each analysis, and specify the measures being plotted. We have also added “data are shown as mean ± SEM” where this information was previously not explicitly stated and clarified when n refers to the number of animals. We believe the revised figure legends now provide clearer guidance for interpretation, and further statistical details are available in the accompanying statistics table. It should also be noted that number of animals used for all experiments can be found in the Method section ‘Mice’.

(3.2) In case experiments were done in a paired manner (e.g., the LC stimulations), individual data points should be shown connected for the different conditions.

We thank the reviewer for this suggestion. While we agree that connecting individual data points is valuable for paired experimental designs, this visualization is not appropriate for the LC stimulation analyses presented here. Specifically, each condition reflects pooled stimulation events selected across animals rather than a single summary value per animal that can be directly matched across columns. As such, individual points in one condition do not map one-to-one onto points in the next condition, making connected visualizations potentially misleading.

Importantly, although the data are presented as pooled event-level measures, the paired structure of the experiment was accounted for in the statistical analyses, such that repeated measurements within animals and the paired nature of the design were included in the relevant comparisons.

(3.3) Numerous analyses involve heart rate measures from the neck EMG during wakefulness. However, it is not specified how, in this case, RR peaks could be detected within the high-activity EMG.

We thank the reviewer for this question. The procedure for heart-rate detection during wakefulness is described in detail in the Heart rate detection Methods section. Briefly, RR intervals were extracted from preprocessed neck EMG recordings using a previously validated approach for mouse sleep studies. To minimize contamination from movement-related EMG activity during wakefulness, R-peaks were not detected within periods extending 100 ms before and 250 ms after detected movement, as these segments were considered too noisy for reliable peak detection. Heart-rate estimates during these excluded periods were subsequently interpolated using surrounding valid RR intervals to preserve temporal continuity and enable analysis of HR dynamics before and after movement episodes.

(3.4) Figure 1b: PSD for RR intervals. The supplementary figure says that 11-minutelong NREMS or 5-minute-long NREMS periods were used. These are very rare events in mice, for which the average bout duration is around 2 min and the cycle length is 10 minutes. The methods do not explain how these bouts were chosen, how many of them were included, and why shorter bouts were not analyzed. Single cases or means?

We thank the reviewer for this important point and agree that additional clarification was warranted. The selection of long NREM (including microarousals) periods was motivated by methodological considerations related to spectral analysis of very slow oscillations rather than by an assumption that these bout lengths are representative of average NREM duration in mice. Because our analysis focused on very-low-frequency dynamics (~1 cycle every 50 s), sufficiently long continuous recordings are required to reliably estimate power at these frequencies and avoid fragmentation-related edge effects that disproportionately affect shorter bouts.

For this reason, shorter NREM episodes were excluded from the PSD analysis, as they do not provide sufficient duration to robustly capture slow-frequency components. We initially compared PSD estimates using NREM periods of at least 11 min (allowing ~10 VLF cycles) and 5 min duration to assess whether the shorter 5 min recordings introduced bias (Supplementary Fig. 1f). While longer periods resulted in higher overall power estimates, the frequency distribution remained highly similar between conditions. We therefore selected 300 s (5 min) as the inclusion criterion for the remainder of the study, as periods exceeding 10 min are uncommon in mice and would substantially limit analyses across experimental paradigms.

To further reduce bias related to bout duration, PSD estimates were weighted by NREM episode length, as longer bouts showed systematic effects on power estimates. For Figure 1, the analysis included 144 NREM-with-MA bouts across 7 animals, and data shown represent group means rather than single examples.

This description is also included in the Methods section/Data Analysis.

(3.5) Figure panel 1c,d: In Panel c, what is plotted?

As stated in the figure legend, it is the cross-correlation between NE and R-R. We have added more description in the figure legend.

A cross-correlation between two signals? If yes, how were these signals chosen per transition? Looks rather like they plot some time course across a transition. What is time point 0?

We thank the reviewer for pointing out that this analysis was insufficiently explained. The analysis shown represents a cross-correlation between the NE and RR signals, performed to assess their temporal relationship across different vigilance-state transitions. Specifically, for each transition type, NE and RR signals were extracted within the corresponding time windows and cross-correlated to determine the strength and timing of their interaction.

In this context, time point 0 (lag = 0) represents perfect temporal alignment between the two signals. Positive or negative lags indicate whether changes in one signal systematically precede or follow changes in the other. Due to methodological differences between the rapid electrical heart signal and the slower fluorescent NE signal, we intentionally avoided overinterpreting fine temporal lead–lag relationships. Rather, the aim of this analysis was to characterize the overall interaction between the two signals across transitions.

Because the relationship between NE and RR was predominantly inverse, negative cross-correlation values indicate that increases in NE are associated with decreases in RR (i.e., faster heart rate), and vice versa. Thus, this analysis served primarily to confirm and extend our other findings by quantifying the interaction between NE and heart-rate dynamics across vigilance-state transitions.

We have revised the descriptive sentence in the Results section to improve clarity and have added a more detailed description of the signals included directly in the figure panel.

(Results, page 7). “Here, cross-correlation analysis revealed a predominantly negative relationship between NE and RR across vigilance-state transitions, indicating that increases in NE were associated with reductions in RR (i.e., faster heart rate; Fig. 1c).”

(Figure 1 legend). “Cross correlation (how strongly and at what temporal offset the two signals covary) between NE and RR during transitions”.

In Panel d, what is measured here? Is the time point of the dotted line a NA trough or the moment of a transition? Show the data with connected lines. Heart rate calculation during wakefulness?

We thank the reviewer for these questions and apologize that this was not sufficiently clear. In panel d, the dotted line indicates the NE trough, not the moment of a vigilance state transition, as specified in both the figure and figure legend. The analysis is aligned to detected NE troughs during sleep and examines the subsequent physiological dynamics, including transitions into wakefulness.

We have updated the sentence in the result section to make it a bit more clear:

(Results, page 7): “To explore the NE-RR relationship across sleep-wake transitions, we examined four progressive sleep-to-wake transitions using the preceding NE trough as the time stamp (time 0):…”

Regarding the suggestion to connect data points, as noted in an earlier response, these analyses are based on event detection, where multiple events contribute from each animal. Thus, each condition represents pooled events across animals rather than a single matched value per animal, making connected-line visualizations inappropriate and potentially misleading. Importantly, the paired structure of the experimental design was accounted for in the statistical analyses.

Heart-rate detection during wakefulness was usually not possible due to movement artefacts in the EMG. In the detection, we excluded periods with movement. Our EMG-based detection approach is described in the Heart rate detection Methods section. Briefly, movement-contaminated periods were excluded from R-peak detection (100 ms before and 250 ms after detected movement), and RR intervals were subsequently interpolated using surrounding valid values to preserve temporal continuity. Because analyses were aligned to NE troughs occurring during sleep, we limited the temporal window to avoid excessive contamination from movement-related noise associated with subsequent wakefulness. We have slightly revised the text to make these points clearer.

Panel C is a cross correlation between NE and RR from the same traces included in the mean traces. x=0 is the NE through like the other figures.

Panel D is the mean traces of NE during transitions with the dotted line at x=0 being the NE though

Yes, but x = 0 for the cross-correlation is not the NE trough. It says something about how aligned NE and R-R are in time (see response further up in (3.5)).

(3.6) The sigma band should ideally be chosen between 10-15 Hz for better consistency with the literature.

We thank the reviewer for this suggestion and agree that consistency in the definition of frequency bands is important for comparison across studies. The sigma range used in the present manuscript was selected to match our previous publications and analyses, thereby allowing direct comparison with our earlier findings on LC-mediated regulation of sleep spindles and infraslow sleep dynamics. Maintaining the same band definition also ensured consistency across the datasets analyzed in this study.

We acknowledge that having a shared definition of sigma power would improve alignment and facilitate comparisons across laboratories. At present, there remains some variability in the exact frequency boundaries used for spindle and sigma analyses across studies and species, although there are ongoing efforts within the sleep field to improve standardization. Importantly, we do not expect that modest adjustments of the sigma-band boundaries would materially affect the conclusions of the present study, as the spindle-related activity of interest lies well within the selected frequency range and the observed effects are broad rather than restricted to a narrow frequency bin.

Moving forward, we aim to follow emerging consensus recommendations where appropriate. To clarify this point for readers, we have added a statement in the Methods section explaining that the sigma band was chosen to maintain consistency with our previous publications.

(Methods: EEG power, page 29). “The sigma band was defined as 8 - 15 Hz to maintain consistency with our previous publications. Modest differences in sigma-band boundaries are not expected to affect the main conclusions.”

(3.7) What are 'extreme LC stimulation frequencies'. The only information available is that stimulations were done for 2s at 20 Hz.

We thank the reviewer for pointing out that this wording was unclear. By “extreme LC stimulation frequencies”, we did not refer to the within-stimulation pulse frequency (which remained constant at 20 Hz for 2 s across all conditions). Rather, we referred to the effective frequency of LC activation at the infraslow timescale, which was progressively increased through the closed-loop stimulation paradigm.

Specifically, stimulations were triggered when NE levels crossed increasingly permissive thresholds during the descending phase of the endogenous NE signal. As thresholds increased over time (from −15 ΔF/F (%) to +5 ΔF/F (%)), stimulations occurred progressively earlier in the infraslow cycle, thereby compressing the oscillatory period and increasing the effective frequency of LC recruitment while preserving the endogenous temporal structure of NE dynamics.

Thus, “extreme stimulation frequencies” refers to the highest rate of repeated LC activations achieved through the closed-loop paradigm, where stimulations became increasingly frequent at the infraslow level rather than changes in the 20 Hz pulse train itself. To avoid confusion, we have revised the wording throughout the manuscript to refer more explicitly to faster infraslow LC activation frequencies or increased infraslow stimulation frequency.

We have added more information in the result section to highlight this better.

(Results, page 8). “We employed a closed-loop paradigm, where LC stimulations (2 s 20 Hz (10 ms) blue laser pulses with a light intensity of 5 mW) were triggered when NE levels fell below increasing thresholds (-15, -10, -5, 0 and 5 ΔF/F (%), Fig. 2a-b, Methods). This approach enabled controlled compression of the infraslow NE cycle by triggering LC activation during the descending phase of the endogenous NE signal, thereby increasing the effective oscillatory frequency while preserving the temporal structure of physiological LC–NE dynamics. This strategy allowed us to test whether heart-rate responses continue to track LC-driven NE fluctuations as the infraslow rhythm becomes progressively faster.”

(3.8) Could the discrepancy between panels 4c, left and right, be due to limited sample size? The whole figure lacks indications of sample numbers, making interpretation difficult.

We thank the reviewer for this comment. We assume the reviewer is referring to the apparent discrepancy between the NE response and heart-rate response following LC suppression (Fig. 4c–d), where LC inhibition induced a clear reduction in NE levels, whereas mean heart-rate responses were less pronounced.

The figure legends state sample sizes and event numbers. Specifically, for these analyses, n = 8 animals (4 Arch, 4 YFP) were included, comprising 48 Arch events and 38 YFP events, our interpretation is that this discrepancy reflects a biological observation rather than a failed manipulation. Specifically, LC suppression robustly reduced NE levels, confirming the effectiveness of the optogenetic intervention, whereas HR did not exhibit a similarly consistent group-level response. However, as highlighted by the correlation analyses, variability in RR responses remained associated with the magnitude of NE suppression, suggesting that heart-rate dynamics still reflected noradrenergic modulation at the individual-response level despite the absence of a strong mean effect.

(3.9) It would be great if Figure 4 j could be more explicitly illustrated. For example, behavioral traces that lead to higher NFR and corresponding changes in RR AUC should be shown for animals with large and small effect sizes. The sample size seems excessively low. Can this explain the difference in slopes compared to Figure 4e, right panel?

We thank the reviewer for this suggestion. To improve the interpretation of Figure 4j, we have now added representative examples illustrating animals with high and low memory performance and their respective RR responses following LC suppression.

We agree that the sample size for this analysis is limited. As noted in the Methods, Figure 4j is based on a secondary analysis of a previously published dataset (Kjaerby, Andersen et al., 2022), where heart-rate measures were retrospectively extracted from EMG recordings. Due to noise-related limitations in RR detection, reliable cardiac measures could not be obtained from all animals, reducing the number of subjects available for this analysis. For this reason, we have deliberately avoided direct statistical comparisons between Arch and YFP animals and instead limited our conclusions to the observed association between RR responses and memory performance across animals.

Regarding the difference in slope compared with Figure 4e, the two analyses are based on different levels of aggregation and address different questions. Figure 4e examines the relationship between NE and RR responses across individual LC suppression events, resulting in multiple observations per animal. In contrast, Figure 4j uses a single mean RR response and a single behavioral outcome per animal. Furthermore, Arch and YFP animals were pooled in Figure 4j to maximize statistical power and because the dataset was not sufficiently powered for direct group comparisons. Consequently, the slopes are not expected to be directly comparable between the two figures.

(3.10) It would be important to show anatomical validation of viral expression in THcre animals and optic fiber positioning.

We thank the reviewer for this comment. Anatomical validation of viral expression in TH-Cre animals and optic fibre placement was performed for these experiments and has been reported previously in Kjaerby et al. Nature Neuroscience paper, from which this dataset was derived. Specifically, viral targeting and fibre positioning were histologically verified as part of the original experimental validation. This is mentioned in the Method section ‘Surgery’: Viral expression and injection sites were validated through immunostaining of perfused brain slices from the experimental animals (see Kjaerby et al. (3) for more information).
